# A very rare case report of glycogen storage disease type IXc with novel *PHKG2* variants

**DOI:** 10.1186/s12887-021-03055-7

**Published:** 2022-05-12

**Authors:** Yongxian Shao, Taolin Li, Minyan Jiang, Jianan Xu, Yonglan Huang, Xiuzhen Li, Ruidan Zheng, Li Liu

**Affiliations:** Department of Pediatric Endocrinology and Genetic Metabolism, Guangzhou Women and Children’s Medical Center, Guangzhou Medical University, Guangzhou, China

**Keywords:** Glycogen storage disease, *PHKG2*, Phosphorylase b kinase, Case report

## Abstract

**Background:**

Pathogenic mutations in the *PHKG2* are associated with a very rare disease—glycogen storage disease IXc (GSD-IXc)—and are characterized by severe liver disease.

**Case presentation:**

Here, we report a patient with jaundice, hypoglycaemia, growth retardation, progressive increase in liver transaminase and prominent hepatomegaly from the neonatal period. Genetic testing revealed two novel, previously unreported *PHKG2* mutations (F233S and R320DfsX5). Functional experiments indicated that both F223S and R320DfsX5 lead to a decrease in key phosphorylase b kinase enzyme activity. With raw cornstarch therapy, hypoglycaemia and lactic acidosis were ameliorated and serum aminotransferases decreased.

**Conclusion:**

These findings expand the gene spectrum and contribute to the interpretation of clinical presentations of these two novel *PHKG2* mutations.

**Supplementary Information:**

The online version contains supplementary material available at 10.1186/s12887-021-03055-7.

## Background

**Fig. 1:**
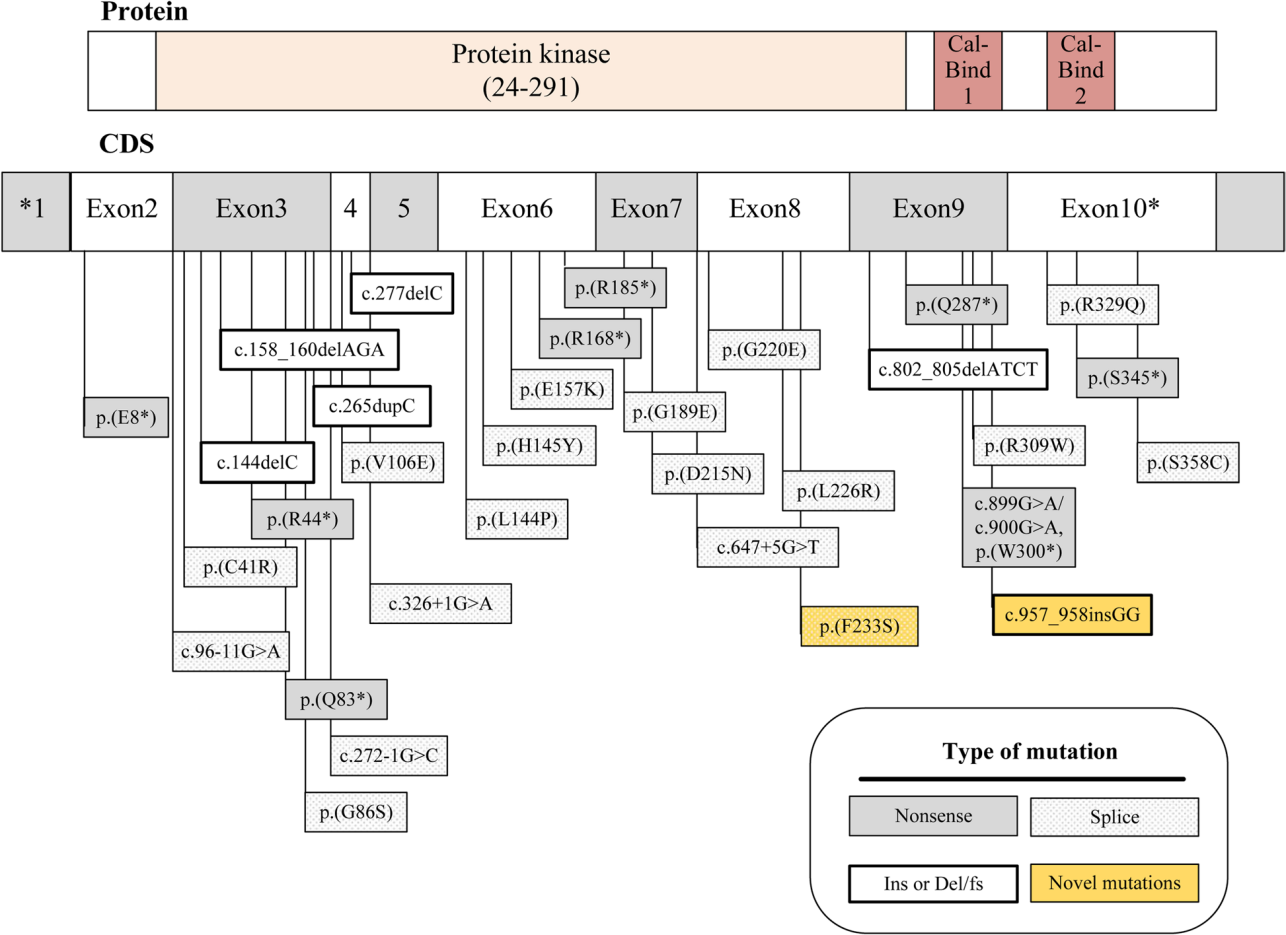
Distribution of mutations among the ten *PHKG2* exons and the protein polypeptide

Glycogen storage disease type IX (GSD-IX) is caused by a deficiency in phosphorylase b kinase (PhK), which is an essential protein kinase regulating the breakdown of glycogen to glucose. PhK consists of four unique subunits encoded by different genes: alpha (*PHKA1* and *PHKA2*), beta (*PHKB*), gamma (*PHKG2*) and delta (*CALM1*) [1, 2]. *PHKG2* is located on human chromosome 16 and contains 10 exons and spans 9.5 kb. Mutations in the *PHKG2* lead to GSD-IXc (OMIM 613027), which is characterized by hepatomegaly, liver fibrosis/cirrhosis, hypoglycaemia, growth retardation, and elevated transaminases, triglycerides and cholesterol [3, 4]. To date, 33 different *PHKG2* mutations have been reported in the literature (Fig. 1) [5]. However, no obvious correlation between mutation severity and liver damage has been found. Allelic truncating mutations in the *PHKG2* have been detected among patients with both cirrhotic and non-cirrhotic liver [6, 7], though the molecular mechanism for these distinct hepatic phenotypes in GSD-IXc remains unknown.

Here, we report a Chinese GSD-IXc newborn with a severe clinical phenotype, including jaundice, progressively elevated liver transaminases and significant hepatomegaly. Two novel mutations in the *PHKG2* were identified by whole-exome sequencing (WES). Specifically, a functional study was performed, showing that the novel mutations destroy protein function. Our findings provide insight into the pathogenicity of these novel variants and benefit clinician work and prenatal diagnosis of GSD-IXc.

## Case presentation

**Fig. 2:**
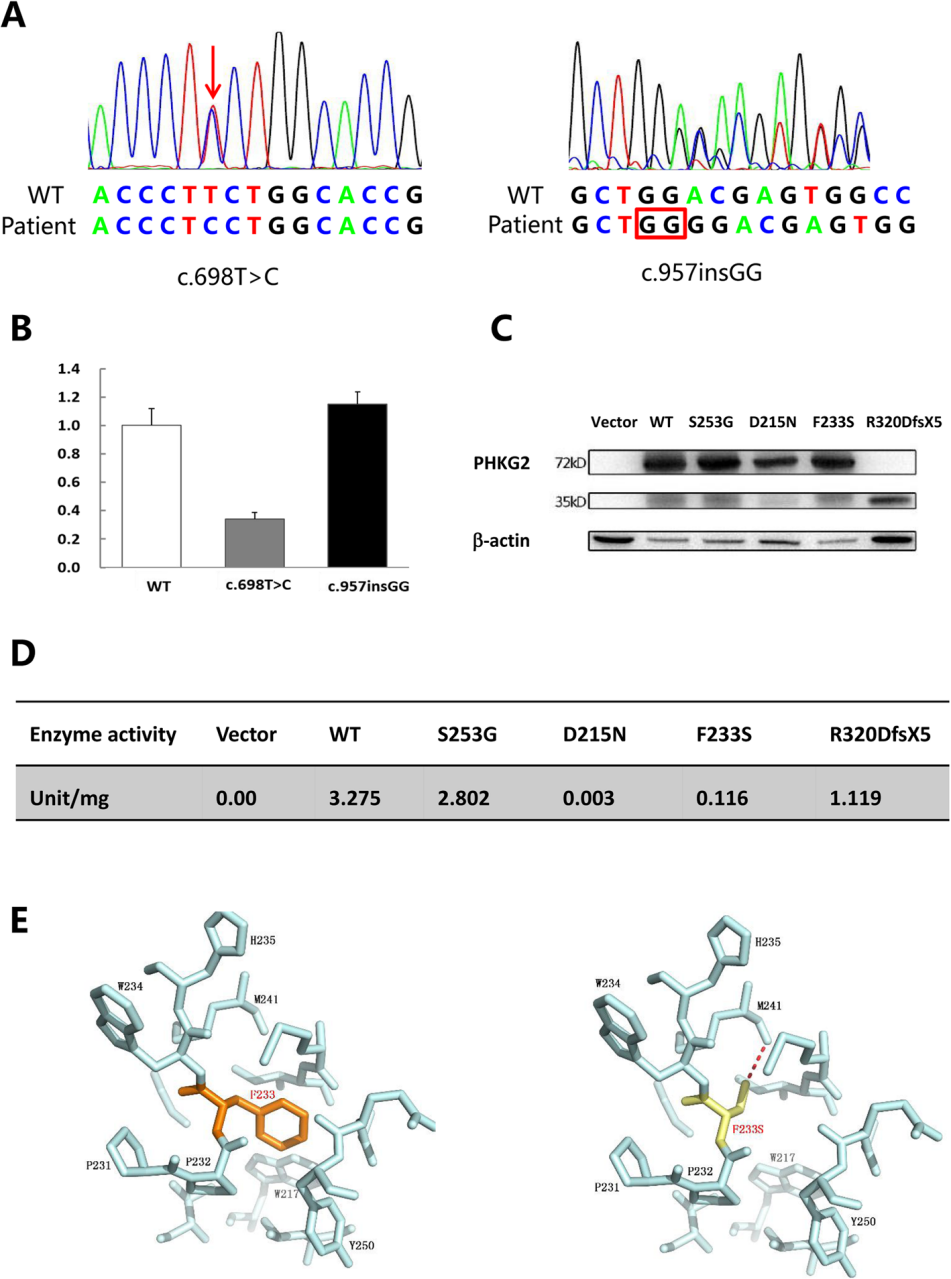
Genotype and modeled structures of PhK gamma catalytic chain protein. **A** WGS discovered the compound heterozygous mutations in patient. The electropherogram depicts missense mutation c.698T>C (F233S) and insertion c.957insGG (R320DfsX5). **B** RT-PCR show *PHKG2* mRNA levels of WT and mutations. **C** Western blots show PhK gamma catalytic chain protein expression of vector, WT and mutations. The molecular mass of the protein fused to EGFP is approximately 72KDa. **D** PhK enzyme activity of WT, polymorphism and mutations. **E** Superimposed structure of native amino acid phenylalanine (orange) and mutant amino acid serine (yellow) at position 233 in PhK gamma catalytic chain protein (2.5Å, PBD code: 2Y7J)

**Fig. 3:**
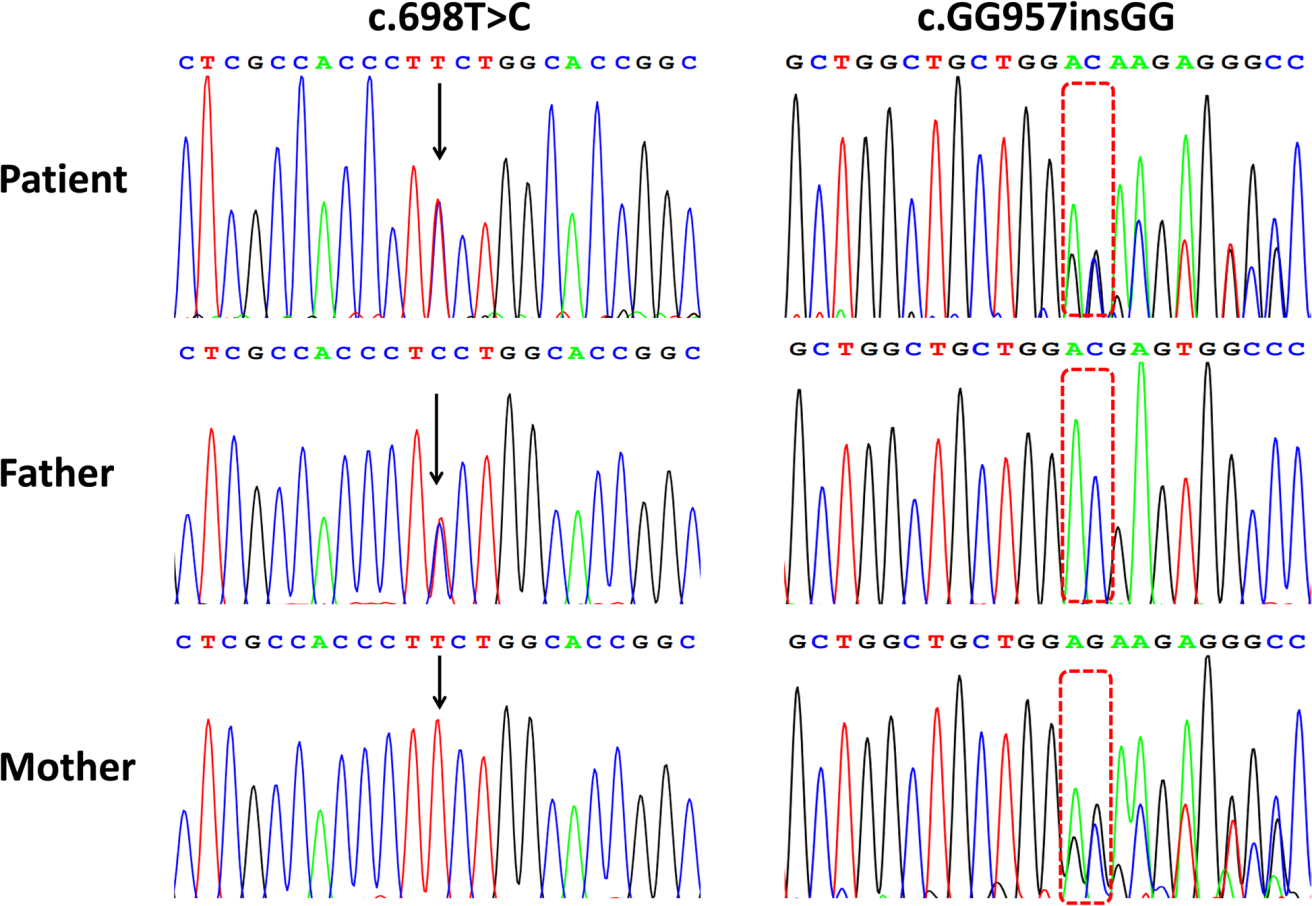
Patient’s mutations on the gene PHKG2 were verified by Sanger sequence. Missensen mutation (c.698T>C) was paternal inheritance whiled insert mutation (c.GG957insGG) was matrilinear inheritance

The patient was born at 40 weeks gestation via a normal vaginal delivery. Her birth weight was 3.0 kg. She exhibited neonatal jaundice, hepatomegaly and elevated serum transaminase from the neonatal period. At the age of 6 months, hypoglycaemia was noted after a 3-h fast. At the age of 10 months, she was brought to our clinical department with a protuberant abdomen caused by massive hepatomegaly. Lactic acidosis was obvious. ALT was elevated at 721 U/L and AST at 910 U/L. Plasma cholesterol and triglycerides were also increased. Uric acid was not elevated. The laboratory examination results of the patient are shown in Table 1. According to the high level of transaminases, GSDVI and IX were suspected [3]. WES was performed to identify phenotype-producing mutations. Two novel variants in the *PHKG2*, the missense mutation F233S (c.698 T > C) and the protein-truncating mutation R320DfsX5 (c.957insGG), were found. The variants were also detected in her parents and confirmed by Sanger sequencing (Figs. 2A and 3). She was given raw cornstarch therapy, and both the hypoglycaemia and lactic acidosis improved; serum aminotransferases decreased from 15 times to 3 times the normal level. Plasma lipid also normalized, but uric acid was slightly elevated. At the age of 10 years, her height and weight were at the 75th centile, though her liver was still enlarged (8 cm below the costal margin).

To evaluate the functional effect of these novel *PHKG2* mutations, a plasmid containing the entire coding region of the human PhK gamma catalytic chain cDNA was constructed. Mutagenesis including the novel mutations, one previously reported mutation (c.643G > A, D215N) [8] and one polymorphism (c.757A > G, S253G) were generated using Quikchange Site-Directed Mutagenesis Kit (Stratagene, La Jolla, CA, USA). HEK293T cells were transiently transfected with these pcDNA3.1-*PHKG2*-EGFP constructs. Polymorphism and mutant agrin were tested by reverse transcriptase-polymerase chain reaction (RT–PCR) analysis and PhK enzyme activity assays according to a previously described method [9]. To analyse the deleteriousness of the protein alteration, virtual models of the *PHKG2* mutations and bioinformatics analysis were performed with PyMOL ^(^™^)^ Molecular Graphics System (Version 1.5.0.3) using the PhK gamma catalytic chain protein crystal structure as a template [2.5 Å, PBD code: 2Y7J].

This report was approved by the Ethics Committee of Guangzhou Women and Children’s Medical Center, China.

## Discussion and conclusions

*PHKG2* mutation is the second most common cause of liver PhK deficiency. PhK is a complex enzyme consisting of four different subunits; the *PHKG2* encodes the testis/liver isoform of the catalytic gamma subunit, which is an active site of PhK enzyme. Hence, PhK deficiency with *PHKG2* mutation is associated with a severe phenotype and has an increased risk of liver cirrhosis [6, 9, 10]. In this case, the patient showed persistent jaundice from the neonatal period, progressive hepatomegaly with hypoglycaemia, lactic acidosis, remarkably high aminotransferases in infancy, and short stature in childhood.

Genetic analysis revealed two novel mutations, F233S and R320DfsX5, in the *PHKG2* (Fig. 2A)*.* To elucidate the destructive effect of these variants, we used HEK293T cells as the host to overexpress either wild-type or mutant *PHKG2* protein, followed by RT–PCR, western blotting and PhK enzyme activity assays. As shown in Fig. 2B, the level of F233S (c.698T>C) mRNA was reduced. The level of full mutant agrin (F233S) expression detected by western blotting (~ 72 kDa) was reduced compared with that of wild-type, which was similar to the result of the reported disease-causing mutation D215N. Another construct encoding the insertion R320DfsX5 was transfected into HEK293T cells, and a truncated protein was observed on western blots (~ 35 kDa), though mRNA levels were unaltered. The PhK enzyme activity of both the F233S and R320DfsX5 mutants was deficient, expressing 3.5 and 34.2% of wild-type, respectively, with the latter presented a higher level of activity than the former.

In addition, 3D protein models revealed structural instability and changes in the secondary structural features of the F233S variant (Fig. 2E). Based on crystal structure data of the PhK gamma catalytic chain protein, residue F233 lies at the loop between helices H8 (residues 214–228) and H9 (residues 238–247), which forms a highly conserved helix-loop-helix domain [11]. Additionally, hydrophobic F233 makes several contacts with amino acids on other helices (W217, P231, P232, W234, H235 and Y250) to form a conjugative effect and stabilize the PhK gamma catalytic chain protein structure. The change from a hydrophobic phenylalanine to a hydrophilic serine causes rearrangement of amino acid side chains and formation of a new hydrogen bond to other helices, destabilizing the folded protein and greatly reducing PhK enzyme activity.

The R320DfsX5 mutation in exon 5 causes a frameshift mutation, which results in the formation of a truncated PhK gamma catalytic chain protein composed of 324 amino acids instead of wild-type with 406 amino acids. To date, nearly half (42%) of reported cases involved truncating mutations, with severe presentations and clinical hypoglycaemic symptoms and progressive liver dysfunction [1, 6]. We reviewed seven truncated mutations (p.R44X; p.H48QfsX5; p.L93SfsX17; p.R168X; p.R185X; p.I268FfsX12; p.Q287X) leading to liver dysfunction or cirrhosis [1, 5, 12] and four truncating mutations (p.H89PfsX13, p.E8X, p.Q83X, p.W300X) that do not result in liver cirrhosis [2, 8, 13], and related PhK enzyme activity is summarized in Table 2. As noted, residues R168, R185, I268, and Q287 belong to the phosphotransferase domain, which plays a key role in the catalytic function of the enzyme [14]. Residues R44 and H48 are located in the phosphorylase kinase domain adjacent to the P-loop motif Gly-Arg-Gly-Val-Ser-Ser-Val-Val (residues 31 to 38), which is a highly conserved component of the ATP-binding site. Residue L93 is located at the end of a series of five tight β strands, which is the hinge region linking the phosphorylase kinase domain and phosphotransferase domain. Hence, we speculate that impairment of phosphotransferase domain functions, the active motif and the hinge region would directly influence PhK catalytic function and decrease PhK enzyme activity, leading to severe liver phenotypes.

In the patient’s recent follow-up, she was a student of grade 4, complied well with dietary advice and had no hypoglycaemia. She still had hepatomegaly, and liver aminotransferases were slightly elevated, but the lipid profile was normal. Her clinical presentation is similar to previous literature reports, and no liver fibrosis or cirrhosis was found by liver ultrasound. Hence, an uncooked cornstarch, low-fat and high-protein diet was recommended. Liver transplantation is rarely needed, except for patients who have significant liver disease.

In conclusion, two novel mutations in the *PHKG2* were identified in this study. Functional and structural analyses proved the pathogenicity of these two novel mutations, which is beneficial for the interpretation of clinically novel mutations in the *PHKG2.* Further, it will help physician recognize this disease and guide the GSD- IXc patient for an uncooked cornstarch diet correctly.

## Supplementary Information

**Additional file 1.** Original data of PHKG2 western blot. (The first land is WT, the second land is S253G, the third land is D215N, the fourth land is F233S, the fifth land is R3200fsXS).

**Additional file 2.** Original data of β-actin western blot. (The first land is Vector, the second land is WT, the third land is S253G, the fourth land is D215N, the fifth land is F233S, the sixth land is R3200fsXS).

**Additional file 3.** Graph of PhK enzyme activity.

**Additional file 4.** Original data of patient and parent’s Sanger sequence.

## Data Availability

All data generated or analysed during this study are included in this published article.

## Abbreviations

GSD-IXc: Glycogen storage disease IXc
PhK: Phosphorylase b kinase
WES: Whole-exome sequencing
RT–PCR: Reverse transcriptase-polymerase chain reaction
ALT: Alanine transaminase
AST: Aspartate transaminase
