# Supplementary figures and images for: A very rare case report of glycogen storage disease type IXc with novel PHKG2 variants
Source: BMC Pediatr. 2022 May 12;22:267. doi: 10.1186/s12887-021-03055-7 (PMC9097106; doi:10.1186/s12887-021-03055-7)

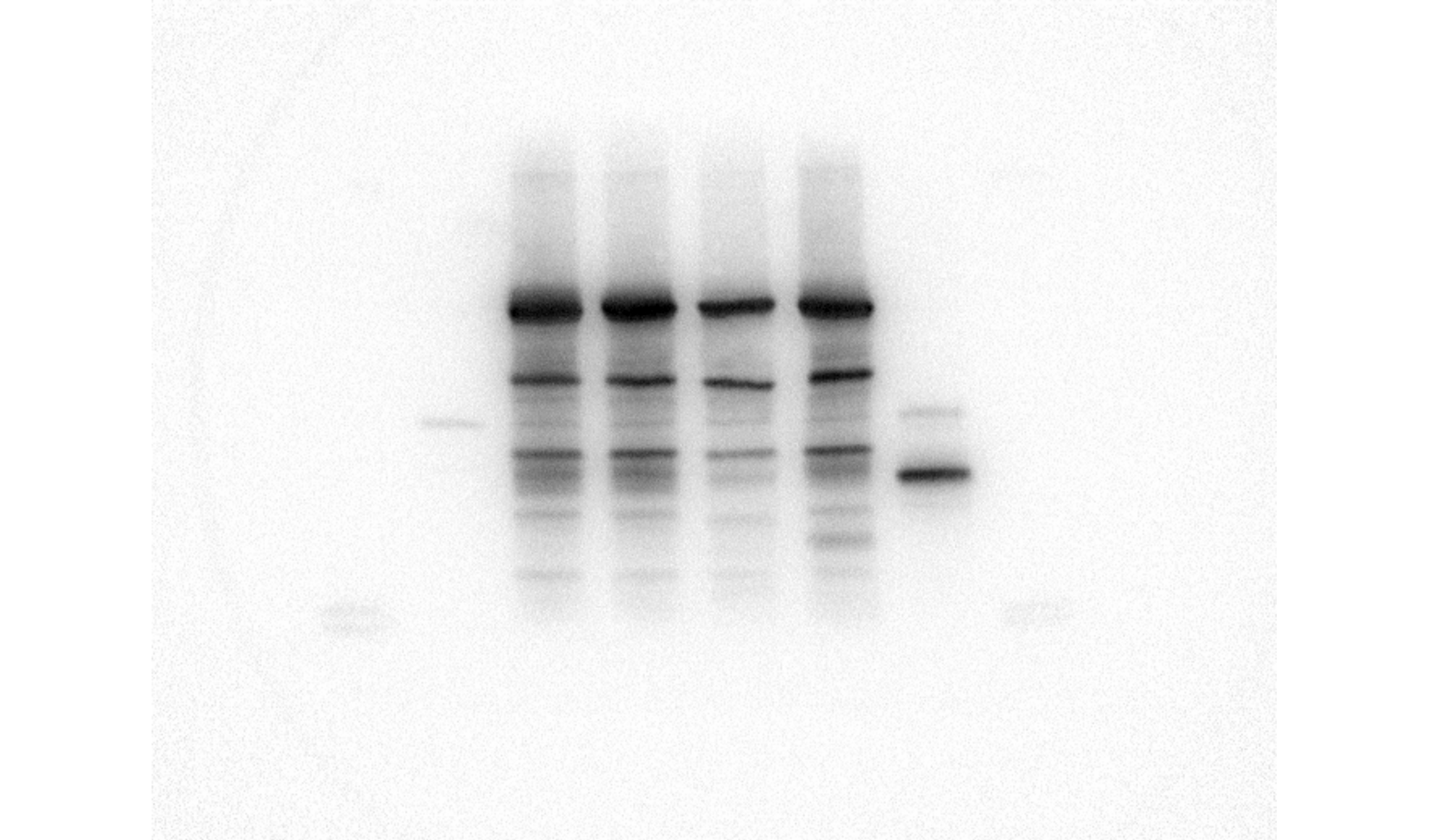

Supplement: Supplementary file 1 — Additional file 1. Original data of PHKG2 western blot. (The first land is WT, the second land is S253G, the third land is D215N, the fourth land is F233S, the fifth land is R3200fsXS). [file 12887_2021_3055_MOESM1_ESM.tif]

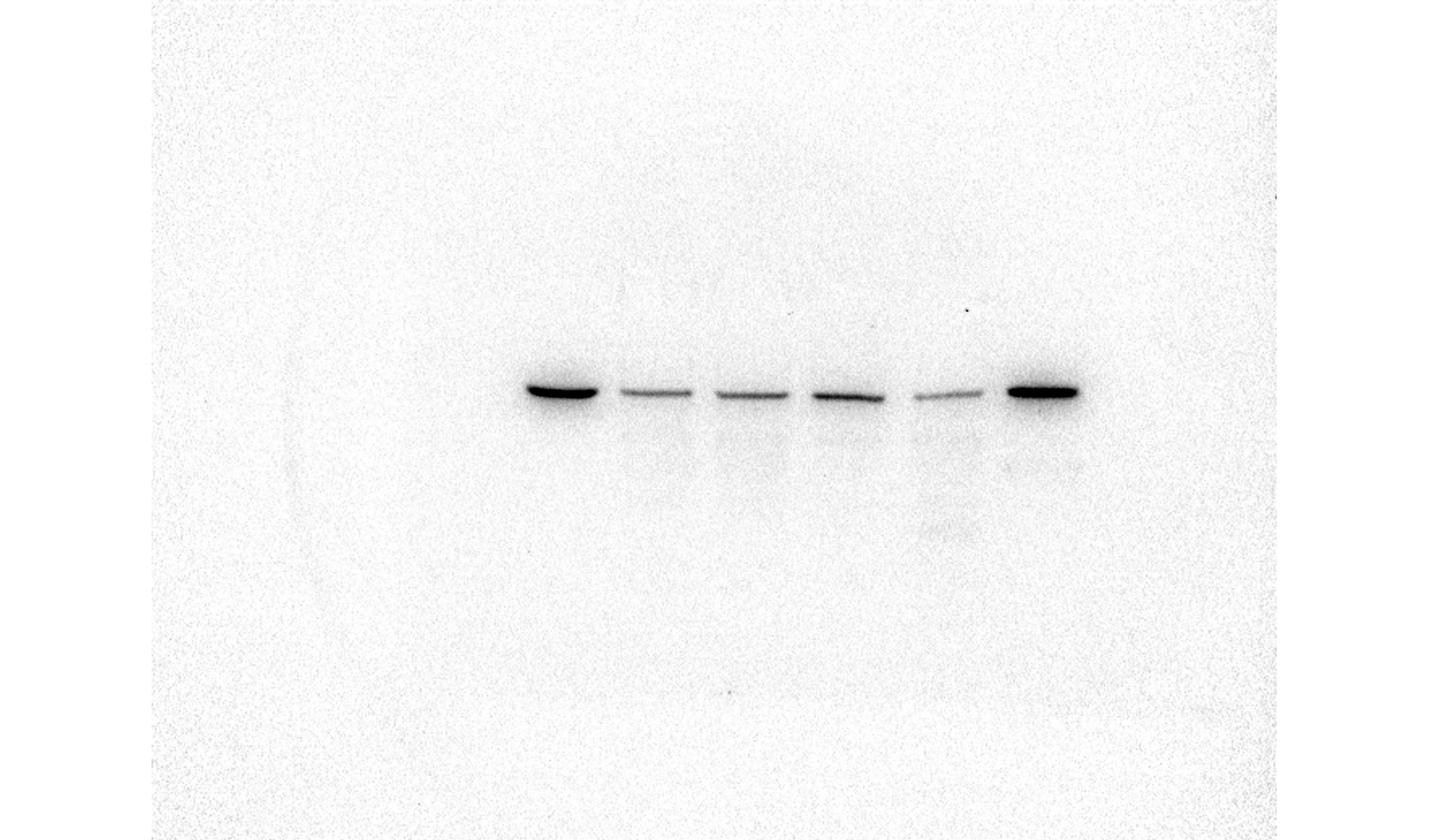

Supplement: Supplementary file 2 — Additional file 2. Original data of β-actin western blot. (The first land is Vector, the second land is WT, the third land is S253G, the fourth land is D215N, the fifth land is F233S, the sixth land is R3200fsXS). [file 12887_2021_3055_MOESM2_ESM.tif]

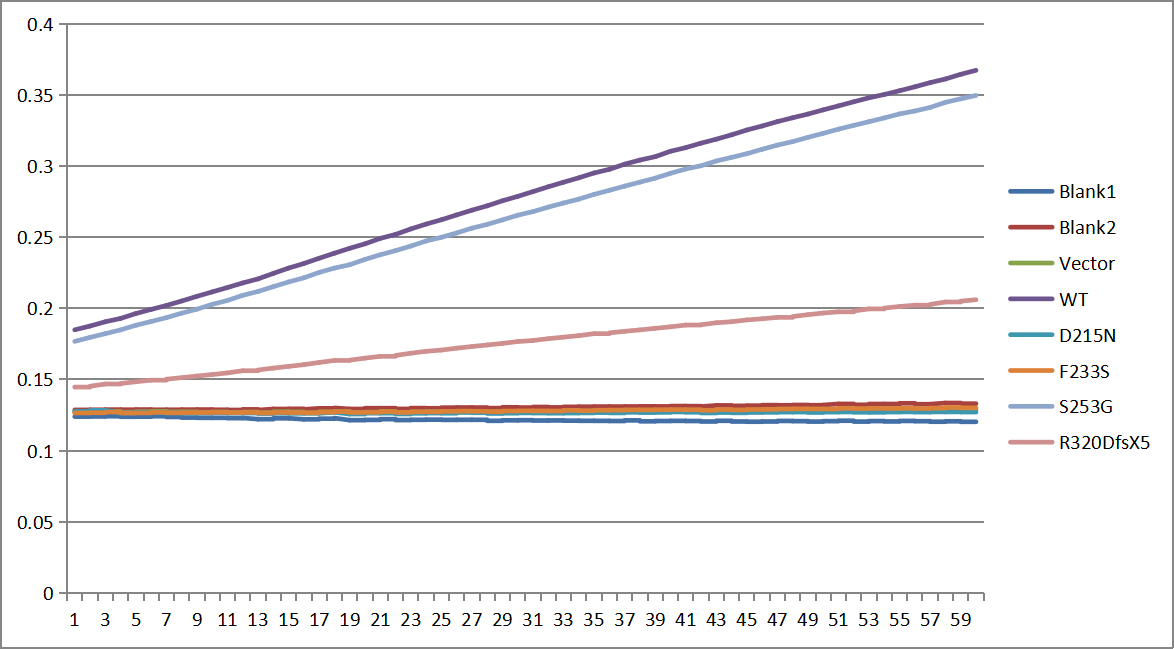

Supplement: Supplementary file 3 — Additional file 3. Graph of PhK enzyme activity. [file 12887_2021_3055_MOESM3_ESM.png]
