# Supplementary material for: A very rare case report of glycogen storage disease type IXc with novel PHKG2 variants
Source: BMC Pediatr. 2022 May 12;22:267. doi: 10.1186/s12887-021-03055-7 (PMC9097106; doi:10.1186/s12887-021-03055-7)

K 288 Patient

c.698T>C(p.F233S)
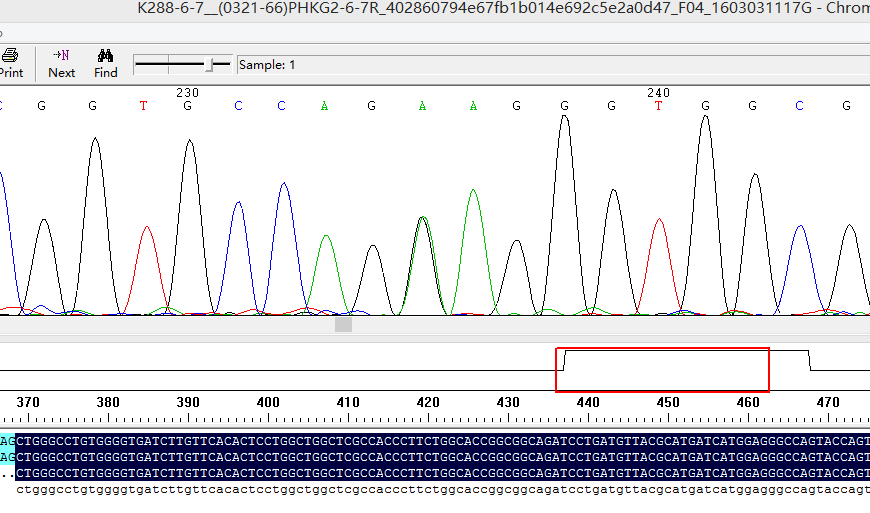


c.GG957insGG(（p.Asp320>ArgfsX）


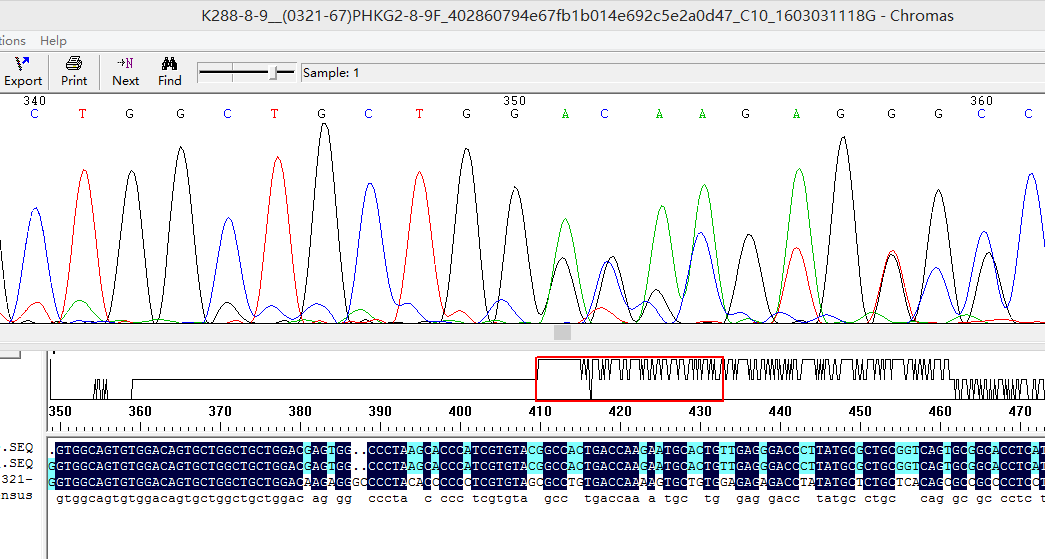


K289 Patient’s father

c.698T>C(p.F233S)


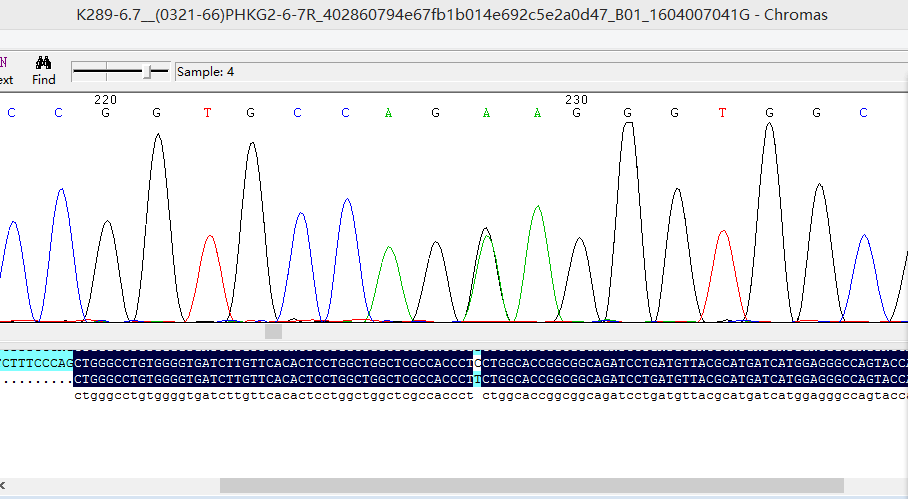


K290 Patient’s mother

c.GG957insGG(（p.Asp320>ArgfsX）


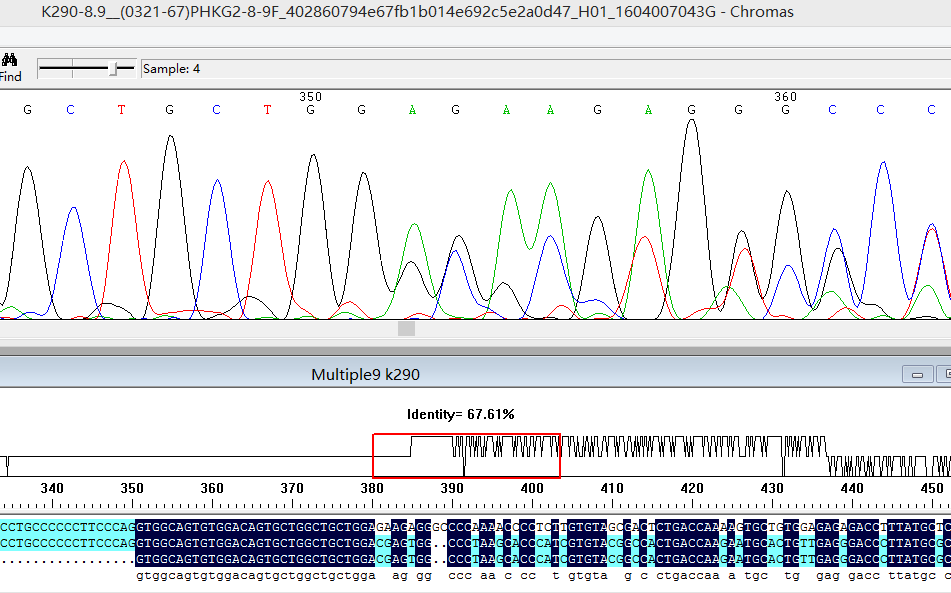

Supplement: Supplementary file 4 — Additional file 4. Original data of patient and parent’s Sanger sequence. [file 12887_2021_3055_MOESM4_ESM.docx]
